# Multiple Bone Fractures in a Patient With Difficult-to-Treat Cushing’s Disease

**DOI:** 10.7759/cureus.29401

**Published:** 2022-09-21

**Authors:** Sara Correia, Diogo Ramalho, Gustavo Rocha, Maria J Oliveira

**Affiliations:** 1 Endocrinology, Centro Hospitalar de Vila Nova de Gaia/Espinho, Vila Nova de Gaia, PRT; 2 Endocrinology and Diabetes, Centro Hospitalar de Vila Nova de Gaia/Espinho, Porto, PRT

**Keywords:** adrenalectomy, hypercortisolism, bone fractures, osteoporosis, cushing’s disease

## Abstract

Osteoporosis at a young age should prompt clinicians to search for secondary causes, namely endogenous Cushing’s syndrome.We report a case of a 33-year-old male with a history of spontaneous fracture of the 12th thoracic vertebra and florid features of Cushing’s syndrome. The physical exam evidenced moon face, facial plethora, muscle atrophy of the upper and lower limbs, and accumulation of abdominal fat. Bone mineral density revealed osteoporosis in the lumbar spine and in the femoral neck. Scintigraphy showed bone fractures in several costal arches, dorsal columns, and sternum. Hypercortisolism was confirmed by blood work. Serum cortisol, adrenocorticotropic hormone and corticotropin (ACTH), and 24-hour urine cortisol values were elevated. Imaging with MRI sellar region was normal and bilateral catheterization of inferior petrosal sinuses was positive. The patient underwent transsphenoidal pituitary surgery (TPS) and a lesion in the right side of the pituitary was identified and resected. Postoperatively, the patient did not meet the remission criteria and we decided to initiate treatment with ketoconazole alongside pituitary radiotherapy. After two years of surgery, the patient presented with recurrent bone fractures, height loss (25 cm), intense fatigue, and difficulty walking without assistance. Due to severe disease, we performed bilateral adrenalectomy, which was essential to control hypercortisolism and improve the patient's quality of life.

## Introduction

Cushing's syndrome, a potentially lethal disorder characterized by endogenous hypercortisolism, may be difficult to recognize, especially when it is mild and the presenting features are common in the general population. It is always necessary to exclude exogenous ingestion of steroids, which can also be a cause of Cushing's syndrome. However, there is a need to identify the condition at an early stage, as it tends to progress, accruing additional morbidity and increased mortality rates [1]. Hypercortisolism leads to a higher incidence of osteoporosis, with bone fractures occurring in 30% to 50% of cases, particularly in the thoracic vertebrae and lumbar vertebrae [2]. Most of the patients with vertebral fractures had multiple fractures [3].

## Case presentation

A 33-year-old Caucasian male patient was referred for suspected Cushing's syndrome. He was diagnosed with a spontaneous fracture of the 12th thoracic vertebra and reported increased weight (8 Kg) in the last two years. He denied a history of hypertension or diabetes mellitus. He was medicated with paracetamol 1000 mg every eight hours and denied taking corticosteroids. On physical examination, the following were observed: moon facies, facial plethora, muscle atrophy of the upper and lower limbs, blood pressure 145/85 mmHg, body mass index (BMI) of 24.5 Kg/m2, accumulation of abdominal fat, and absent purple streaks. The thyroid gland was palpable with no nodules. The hair distribution was normal without ecchymosis or peripheral edema. There was no evidence of acanthosis nigricans. The basal laboratory study revealed cortisoluria of 1190 µg/24h, serum cortisol (8 a.m.) 45 µg/dL, and ACTH (8 a.m.) 77.5pg/mL (Table 1).

**Table 1:** Endocrinological assessment of the patient on admission ACTH: Adrenocorticotrophic hormone, PRL: Prolactin, FSH: Follicle-stimulating hormone, LH: Luteinizing hormone, TSH: Thyroid stimulating hormone, T4: Thyroxine, T3: Triiodothyronine

	Result	Reference values
Hemoglobin	14.8	13.0-18.0 g/dL
Creatinine	1.0	0.7-1.2 mg/dL
Fasting glucose	90	70-105 mg/dL
Total calcium	9.3	8.8–10.2 mg/dL
Albumin	3.8	3.4-4.8 g/dL
Phosphorus	3.6	2.7-4.5 mg/dL
Vitamin D	73	62.5-200 nmol/L
Parahormone	55.7	15.0-65.0 pg/mL
Cortisol (8 a.m.)	45	6.2-19.4 µg/dL
ACTH (8 a.m.)	77.5	0-46 pg/mL
PRL	17.9	4.04-15.2 ng/mL
FSH	3.77	1.5-12.4 mUI/mL
LH	2.3	1.7-8.6 mUI/mL
Total testosterone	6.3	2.8-8.0 ng/mL
Dehydroepiandrostenedione sulfate	>1000	65.1-368 µg/dL
Delta-4-androstenedione	<0.03	0.30-2.63 ng/mL
TSH	0.96	0.27-4.2 uUI/mL
Free T4	0.94	0.93-1.70 ng/dL
Free T3	2.85	2.57-4.43 pg/mL
24-hour urinary cortisol test	1190	55-286 µg/24h

The corticotropin-releasing hormone (CRH) stimulation test established the diagnosis of Cushing's disease (ACTH increased from 185 to 937 pg / mL after 30 minutes). An MRI of the sellar region was unremarkable. The bilateral catheterization of the inferior petrosal sinuses revealed a basal right central/peripheral ACTH gradient >2 and a post-CRH gradient >3 which confirmed the central hypersecretion of ACTH (Table 2).

**Table 2:** Results of bilateral catheterization of the inferior petrosal sinuses IPS: Inferior petrosal sinuses, ACTH: Adrenocorticotrophic hormone

	Right ISP (pg/mL)	Left ISP (pg/mL)	Peripheral vein (pg / mL)
Basal ACTH	1202	117	68
ACTH at 2 minutes	471	118	13
ACTH at 5 minutes	614	443	23
ACTH at 10 minutes	1006	787	284
ACTH at 15 minutes	1273	872	233

**Figure 1:**
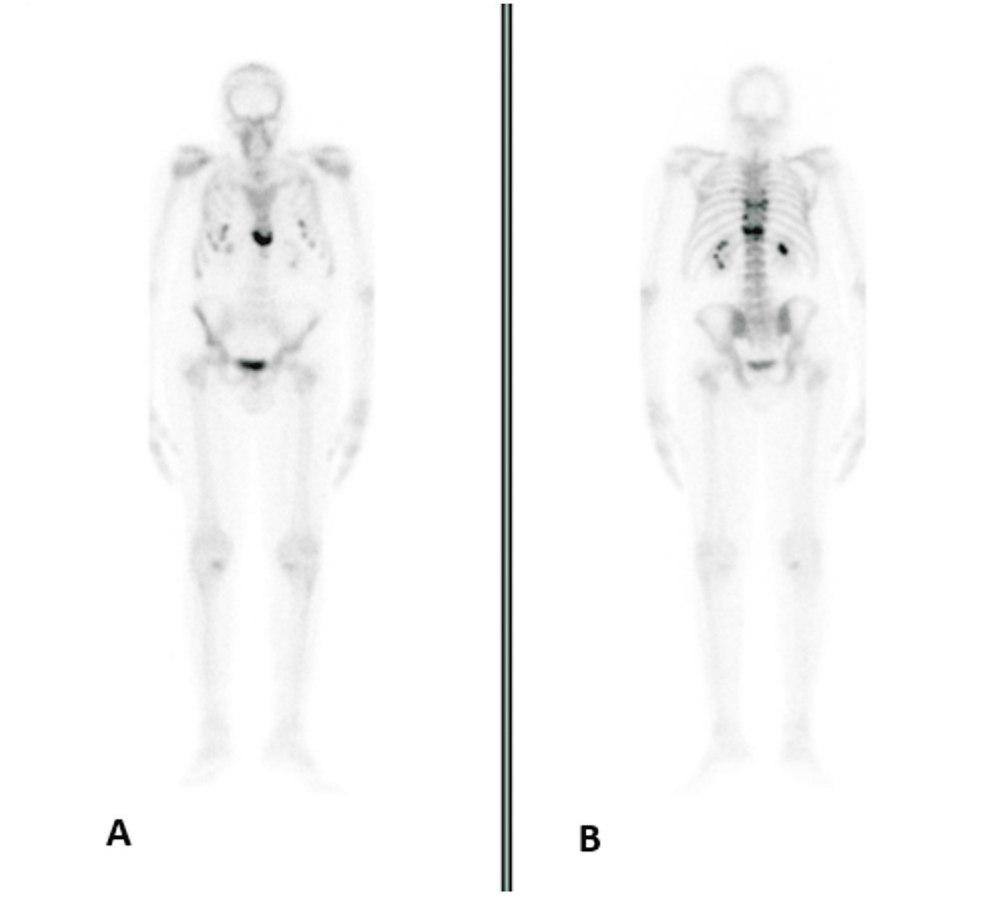
First bone scintigraphy A: Anterior-posterior (AP) view revealed hyperfixation foci of the radiopharmaceutical in the lower end of the sternum. B: Posterior-anterior (PA) view revealed hyperfixation foci of the radiopharmaceutical in the spine, namely in the vertebral bodies D7, D10, D12 and, with linear conformation, in D9 and D11, and also in the anterior extremity of the fifth, sixth and seventh arches bilaterally, consistent with bone fractures.

Dual-energy X-ray absorptiometry revealed total T-scores in the lumbar spine and femoral of -3.8 standard deviation (SD) and -2.0 SD, respectively. Scintigraphy showed bone fractures in several costal arches, dorsal columns, and sternum (Figure 1).

**Figure 2:**
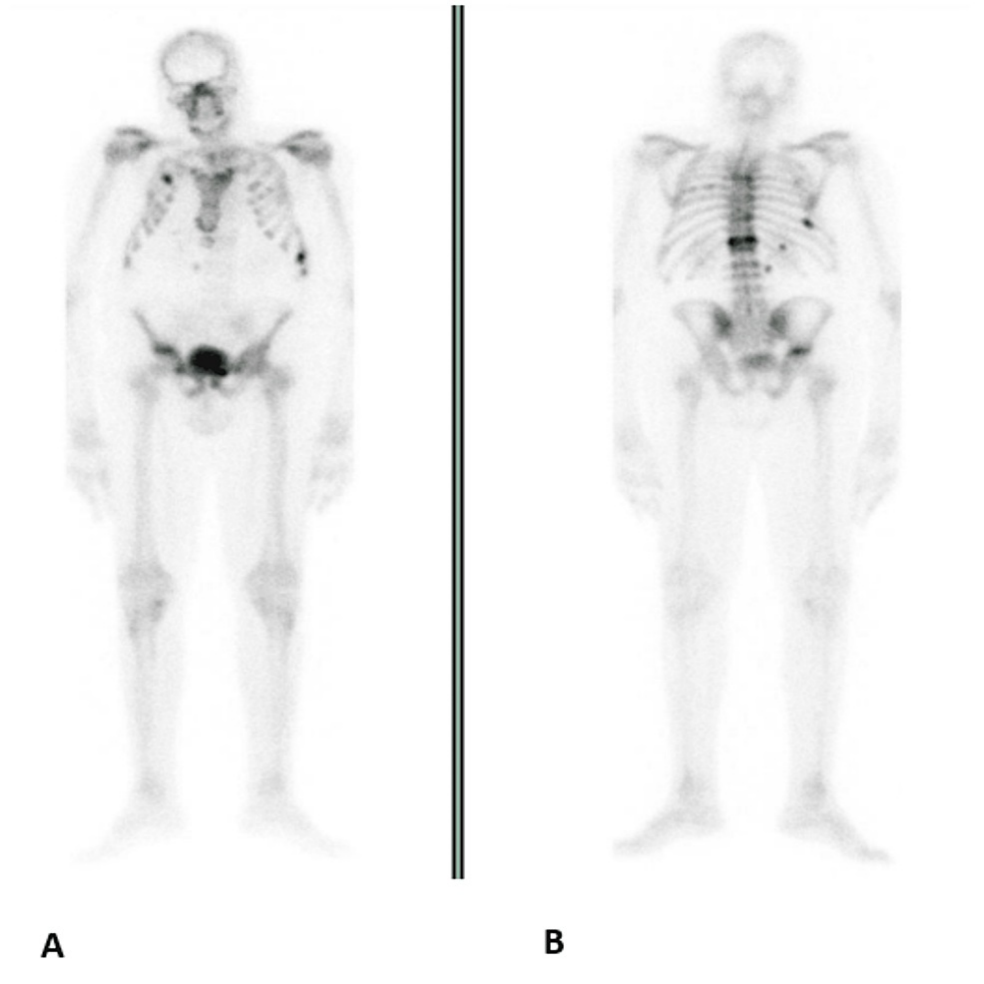
Second bone scintigraphy A: Anterior-posterior (AP) view revealed a significant reduction in the fixation intensity of the radiopharmaceutical in most of the hyperfixation foci described in the first scintigraphy at the level of the spine, anterior extremity of the fifth to seventh left ribs, and sternum. The de novo hyperfixation foci in the costal arches (anterior portions of the second arch and posterolateral of the right ninth arch and anterior extremity of the left eighth arch) are consistent with new bone fractures. B: Posterior-anterior (PA) view revealed marked fixation intensity in D12 and the left superior ramus of the pubis, consistent with new bone fractures.

The osteoporosis was attributed to Cushing's syndrome and other secondary causes were excluded (excessive alcohol use, hypogonadism, hyperparathyroidism, hyperthyroidism, celiac disease, or use of drugs including glucocorticoids or opioids). The patient began treatment with alendronic acid/alendronate, calcium tablets, and vitamin D. The patient underwent transsphenoidal pituitary surgery (TPS) which identified a right lesion on the hypothalamus which was promptly removed. The histological examination was consistent with pituitary hyperplasia and the immunohistochemical study was positive for ACTH. He developed post-surgical central hypogonadism and hypothyroidism. The insulin-like growth factor 1 (IGF-1) levels of 111 ng/mL (94 - 252), exclude growth hormone deficiency. Postoperatively, without remission criteria from hypercortisolism (serum cortisol 8 a.m.= 43.9 µg/dL, ACTH 8 a.m.=79.3 pg/ mL), persisted three months after surgery. The MRI showed no visible adenoma. Given the active disease, the patient was started on ketoconazole at 200 mg/day, which progressively increased up to 800 mg/day. However, it was necessary to reduce the dose due to intolerance (nausea). The patient developed hypertension for which we started valsartan 80 mg/day. Three months later, having maintained hypercortisolemia, he underwent 20 sessions of pituitary radiotherapy (45 Gy), with good clinical tolerance. Despite the strategies that were put in place, the 24-hour urinary cortisol test or salivary cortisol never reached desired levels. The patient's general condition progressively deteriorated with intensified complaints of astenia and new bone fractures (Figure 2), inability to walk without assistance, the need for permanent support in daily activities, and a height loss of 25 cm, all of which prompted the bilateral adrenalectomy.

Histological examination showed diffuse cortical hyperplasia. Post-op follow-up labs revealed cortisol (8 a.m., third day) of 1,0 µg/dL. There was clinical evolution that was very favorable, with the improvement of astenia, cushingoid aspect, and blood pressure. The patient lost 10 kgs and recovered autonomy. Currently, the patient is taking hydrocortisone 30 mg/day divided into two doses (20 mg in the morning plus 10 mg in the afternoon) and fludrocortisone 0.1 mg/day, without clinical or imaging evidence of Nelson syndrome. The MRI performed annually does not reveal any lesion in the sellar area and the fasting ACTH plasma levels have remained below 200 pg/mL. There are no episodes of acute adrenal insufficiency.

## Discussion

Transsphenoidal pituitary surgery is the first line of treatment for Cushing’s disease [1-4]. However, it is not always successful, and the disease may persist in the immediate postoperative period or recur a few years later [5,6]. It allows remission in 60% to 90% of microadenomas, and 50% to 70% of macroadenomas, depending on local invasion and the expertise of the neurosurgeon [4]. In cases of persistent disease, second-line therapeutic options include new surgery, pituitary radiotherapy, or medical therapy. Bilateral adrenalectomy is advocated in patients with active disease when all other treatment options have failed [5,6]. Each of these strategies has advantages and disadvantages [6]. Transsphenoidal re-intervention is preferred in most centers [6], but in this case, it was ruled out by neurosurgery, given the fact that no pituitary lesion was seen in the MRI indicating that there is a lower chance of a cure [7]. A new surgical approach usually entails a higher risk of complications due to the formation of scar tissue and the potential loss of anatomical landmarks [7]. Given the uncontrolled hypercortisolism, the patient was started on therapy with ketoconazole, an inhibitor of adrenal steroidogenesis, and posteriorly pituitary radiotherapy. Usually, the effect of radiotherapy is slow and remission can vary from two to three to 10 years, depending on initial hormone levels [4]. The most recent studies and the largest series in Cushing’s disease in which ketoconazole was used, showed a success rate of approximately 50% [1]. In a dose titration regimen, ketoconazole is usually initiated at 400 mg to 600 mg daily in divided doses. The dose then is up-titrated every three to seven days according to the biochemical response to a maximum dose of 1600 mg daily. Side effects are common and include gastrointestinal symptoms, hepatotoxicity, allergic reactions, male hypogonadism, and gynecomastia. Doses above 1200 mg are less well tolerated [8]. Other drugs such as cabergoline, pasireotide, metyrapone, mitotane, and mifepristone can also be used. Other compounds are currently under research [8].

In this case, given the limited efficacy shown by ketoconazole and radiotherapy in the control of hypercortisolemia, the deterioration of the general condition, and the detection of new osteoporotic fractures, it was decided that bilateral adrenalectomy (three years after the initial diagnosis of Cushing’s disease) would be undertaken for the immediate control of hypercortisolism. We chose not to change alendronate, given that three years had not elapsed since the start of its intake and there are no studies comparing alendronate and zoledronic acid in osteoporosis secondary to Cushing’s syndrome. After the procedure, the phenotypic stigmata regressed and the patient lost 10 kg. The improvement of quality of life after adrenalectomy is reported in 82% to 89% of cases [6].

## Conclusions

When osteoporotic fractures occur at a young age, it is important to exclude secondary causes, such as Cushing’s syndrome. Nowadays, bilateral adrenalectomy is rarely used. It is reserved for patients with severe comorbidities or those refractory to other treatment modalities. Bilateral adrenalectomy rapidly improves symptoms in patients and avoids the long-term complications of elevated cortisol. Specific long-term complications include the development of adrenal crisis and Nelson's syndrome, which requires long-term medical follow-up. 
